# Shared Demands Between Cognitive and Physical Tasks May Drive Negative Effects of Fatigue: A Focused Review

**DOI:** 10.3389/fspor.2019.00045

**Published:** 2019-10-10

**Authors:** Blair Aitken, Clare MacMahon

**Affiliations:** Discipline of Sports and Exercise Science, Department of Dietetics, Nutrition and Sport, La Trobe University, Melbourne, VIC, Australia

**Keywords:** cognitive fatigue, self-control, physiological demands, sport performance, expertise

## Abstract

Over the last decade, there has been increasing interest in the effects of cognitive fatigue on physical performance. This review aims to provide an understanding of these effects with an emphasis on different types of physical tasks requiring different cognitive loads. We specifically focus on understanding the similarity or difference between the cognitive and physical task to help formulate a hypothesis for when there is a decline in the subsequent physical task and when there is not. Taken together, the research shows that endurance performance and perceptual-cognitive skills seem to decline following the completion of a cognitive task, while anaerobic performance remains unaffected. Consistent with the limited-resource model of self-control, the completion of a cognitively demanding task may deplete or inhibit cognitive resources, resulting in impaired performance on a subsequent physical task that relies on similar resources. In other words, physical tasks involving central executive functioning may be significantly more impaired by cognitive fatigue compared to tasks that utilize an all-out strategy. It is recommended that future research continues examining the impact of cognitive fatigue on different types of physical tasks while controlling for other variables.

## Introduction

In our everyday lives we show a remarkable capacity to regulate our thoughts, feelings and behaviors to overcome our desire to stay in bed, say hurtful things to others or procrastinate when we should be working. This ability to exercise deliberate control over our impulses allows us to refrain from behaviors that bring instant gratification and focus on long-term goals that are often paired with more desirable outcomes. Indeed, without the ability to regulate our behavior and engage in the controlled and motivated effort required for goal-directed behavior, life would be merely a series of impulsive actions to serve our immediate needs (Hagger et al., 2010). Despite this ability to regulate ourselves, persistent lapses of self-control are common, especially when we are, among other things, under the influence of alcohol or deprived of sufficient rest to function as we normally would.

Cognitive fatigue refers to a subjective state characterized by increasing feelings of tiredness, low energy levels and decreased motivation, and a decline in performance and aversion to tasks in which associated rewards are low (MacMahon et al., 2014; Van Cutsem et al., 2017). Performing a cognitively demanding activity over prolonged periods induces cognitive fatigue as a result of the strain placed on working memory through continuous or extended use of executive-type mental resources (Hockey and Wietoff, 1993). Often, the completion of one cognitive task will have a negative impact on performance of a subsequent task due to the effects of cognitive fatigue. Indeed, research has demonstrated the negative impact of cognitive fatigue on cognitive function and skill performance in settings such as attention (Boksem et al., 2006) and driving (Lal and Craig, 2001). For example, many road accidents have arisen as a result of cognitive fatigue, usually occurring as a result of sustained performance (Lal and Craig, 2001).

As early as 1891, Angelo Mosso reported that muscle endurance was reduced in two fellow professors of physiology after long lectures and oral examinations, suggesting that cognitive fatigue may also have an impact on physical performance (Mosso, 1891). More recently, work within the domain of exercise science has become increasingly concerned with the effects of cognitive fatigue on physical performance, examining the influence of cognitive fatigue on self-paced and constant load tests of endurance (MacMahon et al., 2014), anaerobic work capacity (Martin et al., 2015), maximal voluntary activation and strength (Rozand et al., 2014), and perceptual-cognitive skill-based performance (Smith et al., 2015a). Several studies have successfully used cognitively demanding tasks as a method of inducing cognitive fatigue, and have shown that completing a cognitive task tends to have a negative impact on a subsequent physical task (MacMahon et al., 2014; Smith et al., 2015a), while others have found no decrement (Martin et al., 2015). In a recent meta-analysis, Van Cutsem et al. (2017) reviewed the findings of 11 studies examining the effect of cognitive fatigue on physical performance. They found an overall decline in physical performance due to cognitive fatigue including decreased time to exhaustion and self-selected power output, and increased time to complete a physical task due to cognitive fatigue.

While research has mainly focused on the physiobiological model, an alternative approach for explaining the impairment during a physical task following a cognitively taxing task is the limited resource model of self-control (Baumeister et al., 1998). This model addresses the capacity to exert self-control by suggesting that acts of self-regulation consume a resource that is limited, leaving people in a state of ego depletion in which the self has few resources. Such resources include executive function which plays an important role in self-regulation as well as effortful choice and active initiative. Due to depletion of these resources the self may be rendered temporarily less able and less willing to function normally or optimally (Baumeister and Vohs, 2007). Muraven et al. (1998) provided initial support for the ego depletion effect. Researchers asked an experimental group to suppress their emotions while watching an evocative video, while the control group freely expressed their emotions. Participants were then asked to hold a spring-loaded hand-grip to exhaustion. They found that, compared to the control group, participants who suppressed their emotions performed worse on the hand-grip task (Muraven et al., 1998). In another study, participants were asked to make a series of choices about consumer products, college courses and class materials, while others thought about the same options but did not make choices. Compared to the no-choice condition, making choices was found to deplete self-control resources leading to less subjective physical stamina, reduced persistence in the face of failure and more procrastination (Vohs et al., 2008). Although there seems to be substantial evidence supporting the claim that cognitive fatigue affects physical performance tasks, there are some conflicting finding. Consistent with the limited resource model, it has been suggested that this disparity between the impact of cognitive fatigue on different physical tasks is due to the difference in cognitive resources required to complete them. If self-control is a finite resource that becomes depleted following a cognitive task, then as the cognitive component of a subsequent physical task increases, cognitive fatigue will have a greater impact on performance (Martin et al., 2015; Van Cutsem et al., 2017).

The aim of this review is to provide an understanding of the effects of cognitive fatigue on physical performance with an emphasis on how this effect plays out for different types of physical tasks requiring different cognitive loads. Although much of the literature has used the term “mental fatigue” as distinguished from physical or muscle fatigue, there has been some disagreement among authors as to whether the term “cognitive fatigue” is more appropriate. For the purpose of this review, the term “cognitive fatigue” will be used as it was deemed more appropriate in the context of the tasks that are typically used which are cognitive in nature and is the component of the manipulation and physical task we are focusing on. Additionally, Englert (2016) makes a distinction between the ego depletion and cognitive fatigue literature based on the length of the cognitive task administered. Englert suggests that cognitive tasks <30 min are in fact self-regulation tasks that lead to ego depletion, as they are not long enough to induce cognitive fatigue. Contrastingly, Hagger et al. (2010) argued that the two are analogous, and ego-depletion may be one way to achieve cognitive fatigue. In agreement with Englert, Van Cutsem et al. (2017) excluded ego depletion studies from their review. This does not seem to provide a satisfactory consideration of the literature, wherein the very same task can be considered ego depletion when engaged in for 20 or 25 min, but cognitive fatigue when engaged in for 30 min. We suggest, rather, and in agreement with Hagger et al.'s suggestion, that ego depletion and cognitive fatigue are degrees of the same state, and the terms may be interchangeable in this context. We are primarily concerned with bringing the findings from the two perspectives together, in a more complete understanding of the phenomenon and when effects are present or absent.

Unlike reviews that have mainly focused on one type of exercise (e.g., endurance; McMorris et al., 2018), or sport (e.g., soccer; Smith et al., 2018), or have taken a broader view of the literature and eliminated studies based on the length of the cognitive task (Van Cutsem et al., 2017), this review on the research into physical performance after cognitive fatigue includes a range of physical tasks. With a specific focus on separating physical tasks into endurance-based tasks (e.g., running and cycling), anaerobic-based tasks (e.g., maximal voluntary contractions), and tasks that rely mostly on perceptual-cognitive skills (e.g., tactical performance). In this way, the primary consideration in examining the studies is with a focus on the cognitive demand required to complete the physical task. Studies used in this review were identified by searching the online databases EBSCOhost, PubMed, Scopus, Web of Science and Google Scholar. Databased were searched by combining the terms (cognitive fatigue OR mental fatigue OR ego depletion) AND (physical OR endurance OR anaerobic) AND (skill OR activity OR performance OR exercise). For the purpose of this review, only studies that have employed a repeated measures crossover design are included. Finally, as this review focuses on the impact of cognitive fatigue on subsequent physical performance, all studies included a psychological manipulation check confirming that the control intervention induced less, or no cognitive fatigue, than the cognitively fatiguing task.

## Endurance Tasks

During a competition, athletes must continuously make decisions about how and when to invest their energy in order to maintain internal homeostasis and avoid early exhaustion. This type of decision making involves a combination of interoceptive (i.e., physiological) and exteroceptive (i.e., environmental) factors that are used to monitor bodily sensations such as pain and muscular fatigue, breathing, thirst, and nutritional needs, body movements and form, and overall effort or feel (Smits et al., 2014; Brick et al., 2015). These factors, along with personal goals and the likely demands of the task are often used to inform the type of pacing strategy adopted by athletes (Smits et al., 2014), and research has shown that pacing ability and performance on a physical task are related. For example, Van Biesen et al. (2016) examined the pacing ability of elite runners with mild intellectual impairment to better understand the role that intellectual capacity plays in running. The pacing ability of well-trained runners with and without intellectual impairment was compared during a 400- and 1,500-meter track race. It was found that runners with intellectual impairments paced their race differently and with greater variability when compared to runners without intellectual impairment, and these constraints on the ability to effectively regulate pace and exercise intensity during a competitive race may be due to the athlete's limited cognitive resources (Van Biesen et al., 2016).

Related to the impact of general cognitive resources on an individual's ability to maintain and achieve an optimal level of performance, the ability to focus self-regulatory cognitions during an endurance task seems to also have a significant effect (Brick et al., 2015; Van Biesen et al., 2016). When an individual completes a cognitively fatiguing task, the self-regulatory resources used to complete the task may become depleted and their overall performance on a subsequent endurance task will be compromised due to an impaired ability to properly utilize self-regulation strategies such as pacing. Within the literature, studies in which the target task is of an endurance nature have mostly found an effect supporting this pattern, with the majority reporting a decrease in physical performance following a cognitive task. For example, Marcora et al. (2009) observed a mean decrease of 15% in a time to exhaustion cycling task performed at 80% of peak power output following 90 min of a continuous computer-based task (the AX-CPT). The AX-CPT involves a sequence of letters being presented one at a time centrally on a computer screen, each for a duration of 300 ms, followed by a 1,200 ms interval. The subject's task is to press the right hand button in response to the target trial and the left for non-target trials. A target trial is defined as one in which the letter A is followed by the letter X. The remaining letters of the alphabet serve as non-target stimuli (Marcora et al., 2009). Similar to Marcora et al., MacMahon et al. (2014) found slower 3 km running times after the AX-CPT. Additionally, following a 30 min Stroop task, swimmers tested by Penna et al. (2017) were 1.2% slower in completing a 1,500- m time trial, and after only 80 trials, Englert and Wolff (2014) reported a decline in performance during an 18 min cycling task compared to a control task. Similar to the AX-CPT, the Stroop task requires sustained attention, working memory and response inhibition. In a typical Strop task, participants are presented with the names of the colors RED, BLUE, GREEN, and YELLOW in a different colored font (e.g., RED presented in blue font) and asked to indicate the color of the font (e.g., blue) rather than indicating the color of the word itself (e.g., RED) by pressing a corresponding labeled key on the keyboard as quickly as possible (Penna et al., 2017). Clearly, then, a range of endurance-based tasks are impaired by cognitive fatigue, however, it is notable that there have been a range of effect sizes from small (*d* =.35; Marcora et al., 2009) to very large (*d* = 1.34, MacMahon et al., 2014).

Endurance tasks that involve a progressively challenging pace of intermittent running, and thus a significant pacing element have also shown the negative effects of cognitive fatigue. For example, Smith et al. (2015a) and Veness et al. (2017) targeted performance on the Yo-Yo Intermittent Recovery Test Level 1 (Yo-YoIR1), a progressive shuttle-run test with an increasing velocity (Veness et al., 2017). In both studies, researchers observed a significant decrease in the distance covered during the Yo-YoIR1 after 30 min of an unmatched Stroop task (*d* =.55, Smith et al., 2015a; *d* =.57, Veness et al., 2017).

Overall, it is clear that endurance tasks requiring a continuous regulation of effort, such as long-distance running, seem to depend at least somewhat on cognitive resources which may become depleted following the completion of a cognitive task. On the other hand, however, it is less clear whether short-duration anaerobic activities requiring all-out strategies depend on the availability of similar cognitive resources and thus will be similarly impaired.

## Anaerobic Tasks

High-intensity anaerobic-based exercise is often characterized by an all-out strategy in which the athlete utilizes explosive power by working maximally from the start of the task and rapidly fatiguing as a result. This type of exercise can be defined as any short-duration, local muscle (e.g., maximal voluntary contraction), or whole-body exercise involving high-intensity bursts of physical activity powered predominantly by metabolic pathways that do not use oxygen (Martin et al., 2015; Van Cutsem et al., 2017). This differentiation from endurance-type tasks that rely on self-regulatory abilities, such as pacing, may have implications for the effects of cognitive fatigue.

Pageaux et al. (2013) found a significant decrease in time to exhaustion in a submaximal knee extensor task after cognitive fatigue, but not on maximum voluntary contraction (MVC). Rozand et al. (2014) also demonstrated that prolonged cognitive exertion does not induce a decrease in the MVC of the knee extensor muscles with no difference in maximal force production or maximal muscle activation between a low, moderate, and high cognitive exertion condition. Other anaerobic tasks have also been used to find comparable results, including a countermovement jump, isometric leg extension, a 3-min all-out cycling test (Martin et al., 2015), and the Wingate Anerobic Test (WANT) (Duncan et al., 2015). Thus, overall, explosive power, voluntary maximal strength and anaerobic work capacity seem to be unaffected by cognitive fatigue. Due to the all-out strategy of these kinds of tasks, high-intensity anaerobically based performance requires fewer decision-making and self-regulatory processes than endurance performance. Therefore, as anaerobic performance is less reliant on cognitive functioning, the completion of a cognitively demanding task may not have an impact on an individual's subsequent performance.

## Physical Tasks That Emphasize Perception and Cognition

Combined with physical performance, an athlete's capability to identify and interpret relevant cues and formulate an appropriate response can have a significant impact on the overall outcome of a competition. Players utilize perceptual-cognitive skills, such as anticipation and decision making to select appropriate technical involvement and movements (e.g., action choice). Therefore, elite players typically display superior perceptual-cognitive skills, particularly in invasion team sports, wherein quick and accurate decisions are emphasized (Smith et al., 2018). Whilst endurance performance relies predominantly on planning and controlling resources, physical tasks that require the individual to read and react to their surroundings may also be impaired under cognitive load and fatigue. For example, Englert and Bertrams (2014) showed that sprint start reaction time decelerated following a depleting transcription task (leaving out the letters e and a) while remaining constant in a control condition (Englert and Bertrams, 2014).

In addition to Englert and Bertrams' findings, a decline in skilled performance using tasks pairing reaction time and decision-making skills has also been found. For example, Smith et al. (2015a), and Smith et al. (2016) assessed the impact of cognitive fatigue on soccer-specific skills with a high decision-making demand. Participants in both studies performed a number of soccer-specific tasks on two occasions following either 30 min of an unmatched Stroop or control task. In addition to the Yo-YoIR1, Smith et al. also asked participants to complete both shooting and passing tests in a perception-action coupled paradigm. The results showed that when cognitively fatigued, participants' shot speed and accuracy declined, as did their passing and ball control (Smith et al., 2015a). Smith et al. also reported a significant impairment in both accuracy of soccer-specific decision-making and reaction-time when viewing film-based simulations of offensive play. That is, participants viewed video footage on a large screen and physically carried out their intended response (i.e., pass or shoot the ball to a particular location) after occlusion of the footage.

Moving beyond the laboratory, this same pattern of a decline in complex perceptual-cognitive movement skills after cognitive fatigue has been shown. For example, Badin et al. (2016), used performance during small-sided soccer games (SSG) as the target performance. Although cognitive fatigue had an unclear effect on physical performance, the quality of technical performance was reduced. While cognitively fatigued, players had a lower percentage of positive involvement, accurate passes, possessions, and successful tackles compared to the control side. Coutinho et al. (2017) similarly showed that after game-based and sports specific cognitive fatigue-inducing tasks players spent less time synchronized in the lateral direction and reduced their speed of team dispersion and contraction during the SSG. A follow up study by Coutinho et al. (2018) improved on the design of the previous study (counterbalancing the fatigue condition between teams), and showed that after cognitive fatigue, there was a greater distance between dyads and a reduction in the teams' stretch index and time spent longitudinally synchronized as measured by GPS. Together, the results demonstrated that whilst cognitively fatigued, a player's ability to recognize important environmental cues and to properly monitor and adjust the performance in a timely manner becomes impaired.

## Expertise Effects

Contrary to previous findings, Schücker and MacMahon (2016) failed to find an effect of cognitive fatigue during an externally paced intermittent running task. Participants completed either an unmatched Stroop or control for 10 min followed by a 20-meter shuttle run test, and while reporting significantly higher perceived cognitive fatigue following the unmatched Stroop task compared to the control, there was no difference in time to exhaustion between conditions (Schücker and MacMahon, 2016). It has been suggested that completing a cognitive task for a duration shorter than 30 min may not be long enough to sufficiently induce cognitive fatigue in participants (Van Cutsem et al., 2017), however, studies using cognitive tasks of 20 min (Coutinho et al., 2017), and as short as 6 min (Englert and Bertrams, 2014) have found the negative effects on performance. An alternative factor that may contribute to the inconsistencies in Schücker and MacMahon's findings may be the level of participant expertise. Martin et al. (2016) found that following a 30-min unmatched Stroop task, professional cyclists displayed greater resistance to the negative effects of cognitive fatigue during a 20-min cycle ergometer time trial when directly compared to recreational cyclists. Martin et al. reported that while the physical performance of the novice cyclists was worse following the cognitive task compared to the control task, there was no difference in performance for the professional group. It may be that the high volume and intensity of training required by professional athletes leads to a greater resistance to the effects of cognitive fatigue. These findings suggest that some types of practiced behaviors may be susceptible to cognitive fatigue, and future research should continue to examine the mediating effect of expertise in the relationship between cognitive fatigue and physical performance.

## Other Moderating and Mediating Factors

The focus of this review is an examination of the existing literature and findings, with a particular focus on the cognitive demands of the physical task, and the hypothesis that the perceptual-cognitive demands of the physical task are a key factor that distinguishes between those studies where there is a decline in performance (i.e., in physical tasks with a perceptual-cognitive demand), and those were there is not (i.e., in tasks with relatively less processing demand). The discussion of expertise as a potential mediating factor raises the issue of other potential moderating and mediating factors of this effect. Two of these, with strong indicators within the literature, are motivation and willpower beliefs. Motivation is a core feature of the psychobiological model, based on motivation intensity theory, which argues that cognitive fatigue decreases an athlete's drive to exercise in two ways: by increasing the perceived amount of effort required for task success (“I am too tired to complete this task”) and by decreasing the perceived reward that can be obtained (“it is not worth completing this task”) (Marcora, 2008). Therefore, rather than an inability to perform due to constraints on cognitive resources as Baumeister et al. (1998) suggests, the effects of cognitive fatigue on a subsequent task may reflect a reluctance driven primarily by motivational limitations (Hockey, 2013). According to the psychobiological model, this increased perception of effort has a negative effect on the performer's motivation for undertaking the subsequent task. This leads the performer to exert less energy, resulting in poorer performance (Marcora, 2008). Regardless of this position, much of the current work that examines the effects of cognitive tasks on physical performance does not assess motivation, or does not do so in depth (i.e., with a one item assessment of degree of motivation to perform the task, e.g., MacMahon et al., 2014).

In more recent development of Marcora's general acknowledgment of motivation, however, Inzlicht et al. (2014) distinguishes between different types of motivation: “want-to” and “have-to,” akin to intrinsic and extrinsic motivation. “Want-to” goals are seen as personally enjoyable and meaningful, and as such feel easier to complete, whereas “have-to” tasks are carried out through a sense of duty or obligation. The view in this modeling is that, following a cognitively demanding task, an individual will be more likely to prefer a task they deem enjoyable or rewarding (intrinsic motivation) than a task they feel they have to do because of external pressure or an introjected goal (extrinsic motivation). Providing support for this view is the finding that monetary incentives can eliminate the performance decrement to a handgrip task after cognitive fatigue (Brown and Bray, 2017). Therefore, this view is that what matters is not only the type of activity but also how that activity is understood by the performer (which can be altered by incentive). When cognitively fatigued, an individual may shift from “want-to” motivation to seeing a subsequent task as “have-to.” Coupled with an increased perception of effort, this motivational change leads to a decrease in drive to complete the task, resulting in lesser performance (Inzlicht et al., 2014). Work in the cognitive to physical sequential task paradigm has begun to examine perception of activities using these ideas, including measurement of motivation, emotion, and attention (e.g., see Stocker et al., 2019), but is in need of greater development in measurement tools (see MacMahon et al., 2019 for assessment of “have to” and “want to” motivation).

A second potential moderator of the effect of cognitive fatigue on physical performance, with support within the social psychology literature, is willpower belief. Specifically, Francis and Jobs (2018) found that some individuals believe willpower is limited and others do not, and that these beliefs drive outcome behaviors and effects. For those who believe willpower is limited, performing a demanding task results in cognitive fatigue, whereas those with a non-limited belief experience an energizing effect. The effects of willpower belief may thus play a role in the degree to which cognitive fatigue influences performance on a task (Francis and Jobs, 2018). For example, in a study conducted by Bernecker and Job (2017), participants were led to either resist a temptation or not, and subsequently perform a Stroop test. Those who believed that willpower is limited performed significantly worse on the Stroop test, while participants with a non-limited belief showed the opposite pattern. While, Brown and Bray (2017) show that motivation may be altered to change the effects of cognitive tasks on physical performance, this remains to be tested in full body tasks. Similarly, it is currently unclear what the effects of willpower beliefs are in the relationship between cognitive fatigue and physical performance tasks. Other potential person-level variables include level of control (e.g., action or control orientation, Gropel et al., 2014) and selflessness (Seely and Gardner, 2003), in addition to the environment, where the presence of others [e.g., social facilitation effects (Sheridan et al., 2019) or of an audience (Epting et al., 2011)] have an effect on physical performance. There is a dearth of research investigating these potential moderators and mediators in the cognitive-physical sequential task paradigm, showing the potential for more nuanced explanations of when cognitive tasks do and do not interfere with the performance of physical tasks.

## Conclusion

Cognitive fatigue refers to a subjective state characterized by low energy levels and changes in mood, such as increasing tiredness and negative affect. Existing literature demonstrates that cognitive fatigue affects performance during a range of sports and physical tasks. The degree to which this effect occurs, however, seems to differ between different types of physical tasks. The current review aimed to understand the effects of cognitive fatigue on physical performance with an emphasis on how this effect plays out for different types of physical tasks requiring different cognitive loads. We specifically examined the literature with respect to the nature of the physical task to provide clues about when and why there is a decline in performance. We found that both endurance performance and perceptual-cognitive skills seem to decline following the completion of a cognitive task, while anaerobic performance remains unaffected. Consistent with the limited-resource model of self-control, existing literature shows that the completion of a cognitively demanding task may deplete or inhibit cognitive resources resulting in impaired performance on a subsequent physical task that depends on similar resources. Importantly, it seems the length of the target task is not a significant factor but rather, that the required amount of executive control or cognition is. While a 20 min endurance task can suffer from the effects of cognitive fatigue (e.g., Martin et al., 2016), so too can a 40 s task, such as a passing drill (e.g., Smith et al., 2015a), if that drill relies heavily on higher order central executive functions, such as recognition and reaction to external cues. In practical terms, tasks in which the athlete must regulate their behavior or that require decision-making are significantly more impaired by cognitive fatigue than physical tasks that utilize an all-out strategy regardless of the length of the task. It is also important to note that while performance on some tasks becomes impaired for more or less elite performers, there are some early indications that expertise, motivation and willpower beliefs play a role in the effect of cognitive fatigue on physical performance (e.g., Inzlicht et al., 2014; Francis and Jobs, 2018; MacMahon et al., 2019).

## Future Directions

While the research investigating the effects of cognitive fatigue on physical performance is now moving toward designs with greater complexity and ecological validity (Coutinho et al., 2017), it is important to recognize that cognitive control manipulations have varied throughout the literature in terms of cognitive demand and duration. For example, a number of studies have employed a 90 min AX-CPT (MacMahon et al., 2014; Smith et al., 2015b), while others have used tasks lasting only 6 min (Englert and Bertrams, 2014). Although the majority of studies have employed central executive inhibition tasks that are thought to activate similar neural pathways (McMorris et al., 2018), the length and type of cognitive task used may influence the degree to which participants become impaired. This highlights that the definition of cognitive fatigue and identification of key components is currently unclear. It is also unclear how long the fatigue that is induced lasts, what is involved in recovery, and what mechanisms or underlying factors are shared with physiological fatigue. Going beyond the existing work we examined that uses the sequential task paradigm, we further speculated about moderators and mediators, identifying some key current candidates. Specifically, the role different types of motivation play (i.e., want-to and have-to; Inzlicht et al., 2014) and will power beliefs (i.e., limited and unlimited; Francis and Jobs, 2018) are currently underexplored, and may point to key mediating or moderating individual variables.

While the focus of this review was on one critical factor, namely the overlap in specific cognitive demand between the cognitive and physical task, it is clearly important that, in addition to exploring the effects of expertise, future research addresses individual difference variables such as personality and beliefs as potential moderators and mediators. A final factor worthy of attention is the role of the environment and setting in which tasks are completed, including the presence of others. This further highlights the issue of transfer and that research is required to confirm whether impairments due to cognitive tasks translate into performance reductions during real competition. There may be factors that influence performance during gameplay that can adjust for cognitive fatigue, such as teamwork and game strategy, that are worth considering Additionally, the cognitive tasks used to induce cognitive fatigue often do not resemble tasks athletes would normally complete prior to competition. Although there is evidence that work is progressing in this direction (e.g., Coutinho et al., 2017), implementing more meaningful cognitive tasks is essential for future research to transition from theory to practice. These advances will help this research to identify the mechanisms responsible for the effects, and most importantly for applied sports science, interventions to combat and minimize the negative impact of these effects.

## Author Contributions

Both authors contributed to the conception of the review. BA organized the database and wrote the first draft of the review. All authors contributed to manuscript revision, read, and approved the submitted version of the review.

### Conflict of Interest

The authors declare that the research was conducted in the absence of any commercial or financial relationships that could be construed as a potential conflict of interest.
